# The British Journals: Natural History

**Published:** 1839-04

**Authors:** 


### NATURAL HISTORY.

### *On an Undescribed Species of Human Intestinal Worm.*

By O'B. BELLINGHAM, M.D., one of the Surgeons of St. Vincent's Hospital.

THE species of intestinal worm which forms the subject of the present communication belongs to the genus ascaris, a genus formed by Linnæus, and adopted by all zoologists since. The following are the characters of the genus:—The body is cylindrical and elastic, more or less attenuated at the extremities; mouth provided with three tubercles or valves; anus a little in front of the posterior extremity in the shape of a transverse cleft. Sexes distinct; genital apparatus in the female having its external opening about the junction of the anterior with the middle third of the body; male organ a double spiculum without any sheath.

Rudolphi has made three divisions of the genus. The first contains those species which are equally attenuated at each extremity. The second division includes those in which the anterior extremity is thicker than the posterior; and the third contains the species in which the posterior extremity is thicker than the anterior. Each of these three divisions he has further subdivided into the species in which the head is provided with lateral membranes, or what he calls winged, and into those in which the head is destitute of these appendages, or what he calls naked. Three species of the genus ascaris inhabit the intestines of the human subject. The ascaris lumbricoides belongs to the first division, in which the body is nearly equally attenuated at each extremity, and to the subdivision in which the head is naked. The ascaris vermicularis belongs to the second division in which the anterior extremity is thicker than the posterior, and to the subdivision in which the head is winged. And the species, the subject of the present communication, (to which I have ventured to give the name Ascaris Alata,) belongs to the third division, in which the posterior is thicker than the anterior, and to the subdivision in which the head is winged. . . . . . .

The third species of the genus ascaris, which occurs in the human intestines, has not, hitherto, been described, (although it would appear to have been already observed in this country,) as yet I have met with it only once. It belongs to the third division in Rudolphi's arrangement, and to the subdivision in which the head is winged. From the distinctness of the lateral membranes of the head, I have given it the name of Ascaris Alata.

The two specimens which I possess are females; they are about three inches and a half in length; the greatest diameter, posteriorly, three quarters of a line; the shortest diameter, anteriorly, half a line. The body is cylindrical, of a dirty yellowish colour, marked with the four longitudinal lines, and with the transverse very close striæ, which we find in the other species; the anterior extremity is inflexed, the position straight. The anterior extremity is provided, on each side, with a very distinct semitransparent membrane, a line and a half in length, narrower anteriorly than posteriorly, which commences on each side at the tubercles of the mouth, and together give this part of the body a triangular shape. The three tubercles, or valves, which surround the orifice of the mouth, are prominent, small, but distinct; the diameter of the body very gradually increases from the anterior to the posterior extremity; its termination is conical, and at the very point is a small, dark-coloured spot. The anus is a little in front of the posterior extremity, on the abdominal surface; it runs transversely; is slightly curved, the convexity anteriorly; it is provided with two lips, as in the other species of the genus.

The only instance in which I have, as yet, met with the Ascaris Alata was on the occasion of my prescribing for a child, aged about five years, who exhibited symptoms of worms. I ordered some vermifuge medicine, and desired, in case any worms were voided, that they should be kept. A day or two afterwards, the specimens, from which I have taken the above description, and which had been expelled by the medicine, were brought to me; they were dead when I received them, and I could not learn that the child ever passed any since.

Although this species of intestinal worm had not been previously named or described, it would appear that one, closely resembling it, had been, as I have said, already observed in this country. In the fourth and fifth volumes of the "Transactions of the Association of the King and Queen's College of Physicians" is contained a very interesting case, in which great numbers of insects, and their larvæ, were passed by a female residing in the county of Cork: in several instances the Ascaris Lumbricoides, and a species similar in many points to the one I have described, were also voided by the same individual. Dr. I. V. Thompson, who examined and figured it, says "it resembles the ascaris of the cat, but may probably be a distinct species."

This species, the Ascaris Alata, is very distinct from the Ascaris lumbricoides of the human subject: in general appearance it is not unlike the Ascaris Mystax, which inhabits the stomach and small intestines of the cat; it differs, however, in having a greater diameter posteriorly than anteriorly, and in the lateral membranes of the head being broader in the Ascaris Mystax than they are in the species under consideration. There are some minor points in which they also differ which will be observed if we contrast the characters of the two species.

*Dublin Medical Press. Feb.* 20, 1839.

[The above interesting communication is extracted from the seventh number of a new journal, now published every Wednesday in Dublin. It contains some valuable lectures and original papers, and promises to be a work of much interest and value to the profession in Ireland. It consists of a large sheet of sixteen pages, is stamped for the convenience of transmission by post, and the price is Sixpence.]

